# Cognitive function in Prefrail and frail community-dwelling older adults in China

**DOI:** 10.1186/s12877-019-1056-8

**Published:** 2019-02-27

**Authors:** Lina Ma, Li Zhang, Fei Sun, Yun Li, Zhe Tang

**Affiliations:** 1 Department of Geriatrics, Xuanwu Hospital, Capital Medical University, Beijing, 100053 China; 2 Beijing Geriatric Healthcare Center, Xuanwu Hospital, Capital Medical University, Beijing Institute of Geriatrics, Key Laboratory on Neurodegenerative Disease of Ministry of Education, Beijing Institute for Brain Disorders, China National Clinical Research Center for Geriatric Disorders, Beijing, 100053 China

**Keywords:** Frailty, Cognition, Cognitive frailty, Older adults

## Abstract

**Background:**

Physical frailty, characterized by reduced physiologic complexity and ability to cope with stressors, is closely associated with cognitive impairment, which increases the risk of poor clinical outcomes. To better capture the association between frailty and cognitive impairment, a new construct, cognitive frailty, has been proposed. Cognitive frailty is a clinical condition characterized by the simultaneous presence of physical frailty and cognitive impairment. There is little evidence on the relationship between physical frailty and cognition, as well as cognitive frailty, in Chinese older adults. We aimed to elucidate whether physical frailty is associated with cognitive impairment in an older Chinese population.

**Methods:**

Data were obtained from the China Comprehensive Geriatric Assessment Study. The sample comprised 3202 community-dwelling adults, aged 60 years and older, from seven Chinese cities. Physical frailty was assessed using a modified, four-item version of the Fried criteria, according to frailty phenotype. Cognitive function was assessed using the Mini-Mental State Examination (MMSE).

**Results:**

The prevalence of physical frailty, prefrailty, cognitive impairment, and cognitive frailty was 9.9, 33.9, 7.5, and 2.3%, respectively (weighted: 8.8, 33.8, 6.5, and 2.0%). The prevalence of the combination of prefrail/frail and cognitive impairment was 5.1% (weighted 4.5%). Frail participants performed worse on global cognition and all cognitive domains than robust and prefrail participants. The MMSE total score was positively correlated with walking speed and negatively correlated with age and frailty. A multivariate logistic regression revealed that after adjusting for age, gender, education level, living area, and chronic diseases, frailty, exhaustion, slowness, and inactivity were significantly associated with poor global cognition.

**Conclusions:**

The standard prevalence of physical frailty, prefrailty, cognitive impairment, and cognitive frailty in community-dwelling older adults in China was 8.8, 33.8, 6.5, and 2.0%, respectively. Frailty, exhaustion, slowness, and inactivity were significantly associated with poor global cognition.

## Background

Aging is associated with both physical and cognitive decline. Frailty is a medical syndrome characterized by diminished physical function and reduced age-related physiologic reserve leading to decreased resistance to stressors and increased vulnerability to disability, morbidity, and mortality [1, 2]. Therefore, frailty has become a major public health issue. Since frailty better captures variations in health risks than chronological age [3], it has been suggested as a better predictor of health and well-being and might be a better indicator of adverse outcomes among older adults [4]. However, despite its wide use in clinical practice, there is no consensus on the definition of frailty. The most common approach to measure frailty is the Fried frailty phenotype with biological underpinnings encompassing five components including unintentional weight loss, exhaustion, weakness, slowness, and inactivity [1]. Another important measurement is Rockwood’s frailty index, which considers frailty in terms of deficit accumulation [5, 6]. The close association of frailty and cognitive impairment (CI) increases the risk of mortality in later life. It is estimated that 115 million people worldwide will have dementia by 2050. Declined cognitive reserve leads to mild cognitive impairment (MCI) and dementia, which runs parallel to the course of physical frailty. CI is associated with increased risk of walking speed decline and future frailty [7, 8], whereas frailty predicts cognitive decline/incident dementia [9, 10]. Lee et al. found that the association between frailty and mortality was moderated by baseline cognitive status [11]. As early detection and intervention development to prevent poor clinical outcomes are becoming increasingly important, taking cognition into account may allow for better prediction of adverse outcomes of frailty in later life.

Although a growing number of studies are now focusing on the relationship between frailty and CI, literature has generally considered them as two different entities. Given the many risk factors and underlying mechanisms common to physical frailty and CI [12], the International Academy on Nutrition and Aging and the International Association of Gerontology and Geriatrics have proposed a new construct, “cognitive frailty” (CF)—a clinical condition that describes the simultaneous presence of physical frailty and MCI [13]—providing a framework for research to identify individuals with CI caused by non-neurodegenerative conditions. Although there is no universal consensus regarding CF, it has been used in many recent studies, where it has been associated with greater risk of adverse outcomes [12, 14]. With aging, older persons on a CF trajectory exhibit the greatest burden of nursing home admission and disability [15].

So far, there is still little evidence on the relationship between physical frailty and cognition in Chinese older adults. We previously reported a 3.3% prevalence of CF in mainland China with the definition of CF defined by frailty index and the Mini-Mental State Examination (MMSE) and identified its related factors [16]; however, whether physical frailty was associated with worse cognition is still unknown. Thus, we conducted this study to further elucidate whether physical frailty is associated with CI in an older Chinese population.

## Methods

### Study design

Data were obtained from the China Comprehensive Geriatric Assessment Study (CCGAS), and stratified, multiple-stage, random, and cluster sampling methods were used to recruit community-dwelling participants in China aged 60 years and older between 2011 and 2012. In the first stage, three cities (Beijing, Xi’an, and Harbin) were chosen from the northern cities, and four cities (Chengdu, Chongqing, Changsha, and Shanghai) were chosen from the southern cities. These selected seven cities represented the six main regions of China [17, 18]. In the second stage, older adults residing in different urban and rural areas were selected, using age group and sex ratios based on information about the population composition from the Sixth National Census (2010). Of the 6867 community-dwelling older adults, 3202 individuals without a history of dementia and with a physical frailty assessment and MMSE data were included. Compared to the 3202 included participants, those excluded were older (71.94 ± 8.03 vs. 70.14 ± 7.08 years, *p* < 0.001), had worse cognitive function (MMSE score: 25.85 ± 5.22 vs. 26.71 ± 4.69, *p* < 0.001), were more disabled (11.9% vs. 4.2%, *p* < 0.001), and had more chronic diseases (≥ 2: 64.4% vs. 53.9%, *p* < 0.001).

### Data collection

The clinical and demographic variables related to each subject were collected using a questionnaire administered in a face-to-face interview by trained staff, along with a physical examination. The physical examination was conducted at home or at a central location. Data gathered included the sociodemographic characteristics (e.g., gender, age, education level, marital status, income), anthropometric measurements (e.g., height, weight), health status, personal habits, mental health (e.g., cognition, depression) and variables regarding frailty assessment were also collected. Waist-to-hip ratio (WHR) was calculated as waist measurement divided by hip measurement. Body mass index (BMI) was calculated by dividing the weight in kilogram by height in meter squared and BMI cutoffs were based on Asian adjustments. Income was defined as the amount of money received monthly from an employer or an individual’s from family (for those without a job). Participants were considered to have a medical condition if they had a self-reported history of chronic disease diagnosed by a doctor. Functional ability was assessed based on the capacity of individuals to perform activities of daily living (ADL) and instrumental activities of daily living (IADL). The list of activities consists of 14 items (Eating, grooming, dressing, transferring in and out of bed, bathing, walking inside the house, using the toilet, cooking, managing finances, driving or using public transportation, shopping, walking 250 m, cutting toenails, and climbing stairs), and an individual’s performance on each item is classified as independent, partially dependent and completely dependent, scored 1, 2, and 3, respectively.

### Cognitive assessment

Cognitive function was assessed using a 30-question MMSE; each correctly answered question was awarded one point, whereas incorrect or no answers were awarded zero points. The total score ranged from 0 to 30 points. A recent study in a Chinese population showed age-, gender-, education-, and residence-specific reference norms for the MMSE [19]. The thresholds for those who were illiterate, or attended at most primary school, middle school, or university were ≤ 17, ≤ 20, ≤ 22, and ≤ 24, respectively. Participants who scored below the threshold value for their education group were classified as CI [20]. MMSE assesses ten different cognitive domains: comprehension (range 0–3), reading (range 0–1), naming (range 0–2), drawing (range 0–1), writing (range 0–1), repetition/registration (range 0–3), orientation to time (range 0–5), orientation to place (range 0–5), recall (range 0–3) and attention (range 0–6).

### Frailty assessment tool

Physical frailty was assessed using the modified, four-item version of the Fried criteria (weakness was not considered), according to the frailty phenotype derived from the Cardiovascular Health Study. The four items are unintentional weight loss, self-rated exhaustion, slow walking speed, and inactivity. Weight loss was defined as BMI less than 18.5 kg/m^2^. Exhaustion was indicated by a self-response as “yes” to “Is it hard for you to start on new projects” and “no” to “Do you feel full of energy” from the Geriatric Depression Scale. Inactivity was defined as exercising for < 3 h/week over the past 12 months. Walking speed was evaluated with a 20-m walking test. Slowness was defined as the lowest quintile of the walking speed, adjusted for gender and standing height of the participants: < 0.67 m/s for males with height > 166 cm, < 0.65 m/s for males with height ≤ 166 cm, < 0.63 m/s for females with height > 155 cm, and < 0.57 m/s for females with height ≤ 155 cm. The fulfillment of two or more of the above criteria on the frailty scale was classified as frail, the fulfillment of one was considered prefrail, and no criterion was nonfrail.

### CF criteria

Participants positive for both Fried frailty phenotype and cognitive assessment were classified as having CF.

### Statistical analysis

All statistical analyses were performed using the SPSS for Windows Version 11.5 (SPSS Inc., Chicago, IL). The chi-squared test was used to compare categorical data, which were expressed as numbers and percentages. Continuous data were analyzed using 1-way analysis of variance and were expressed as means and standard deviations. Spearman’s rank correlation coefficient was used to determine correlations. The standard rates calculated using the national standard population composition ratio as at the Sixth National Census (2010). A forward stepwise logistic regression was conducted to explore the association between the related factors as independent variables and CI as the dependent variable. Hypothesis testing was two-sided, using a level of significance of 0.05.

## Results

According to the Fried criteria, of the 3202 older adults without dementia, 317, 1087, and 1798 participants were frail, prefrail, and robust and the prevalence was 9.9, 33.9, and 56.2% (weighted: 8.8, 33.8, and 57.4%), respectively. A total of 241 participants (prevalence 7.5%, weighted: 6.5%) were cognitively impaired. The prevalence of CF was 2.3% (weighted 2.0%). A total of 164 participants were both prefrail/frail and CI (5.1%, weighted 4.5%). Compared to robust adults, both frail and prefrail older adults were older, had lower BMI, slower walking speed, and performed worse on ADL and IADL. Older adults living in rural areas or those with low monthly incomes had a higher prevalence of frailty (Table 1).

**Table 1:** Characteristics of robust, prefrail and frail participants

	Robust	Prefrail	Frail
Number (%)	1798(56.2)	1087(33.9)	317(9.9)
Age (years)	69.25 ± 6.71	70.36 ± 7.00*	74.39 ± 7.83*Δ
Gender (male, %)	798(44.4)	468(43.1)	140(44.2)
Area (rural, %)	551(30.6)	345(31.7)	154(48.6)*Δ
Income (yuan)/month	2463.04 ± 1864.63	2303.35 ± 1576.80	1829.18 ± 1710.89*Δ
BMI (kg/m^2^)	24.31 ± 3.16	23.66 ± 3.56*	22.67 ± 3.98*Δ
WHR	0.8720 ± 0.0636	0.8724 ± 0.0585	0.8718 ± 0.0695
Walking speed (m/s)	1.01 ± 0.32	0.77 ± 0.34*	0.60 ± 0.21*Δ
ADL	7.01 ± 0.10	7.05 ± 0.42*	7.38 ± 1.15*Δ
IADL	7.15 ± 0.78	7.34 ± 1.27*	8.76 ± 2.97*Δ

*Abbreviations*: *BMI* body mass index, *WHR* waist hip ratio, *ADL* activities of daily living, *IADL* instrumental activities of daily living

^*^P < 0.05, compared to robust group; ^Δ^*P* < 0.05, compared to prefrail group

**Fig. 1:**
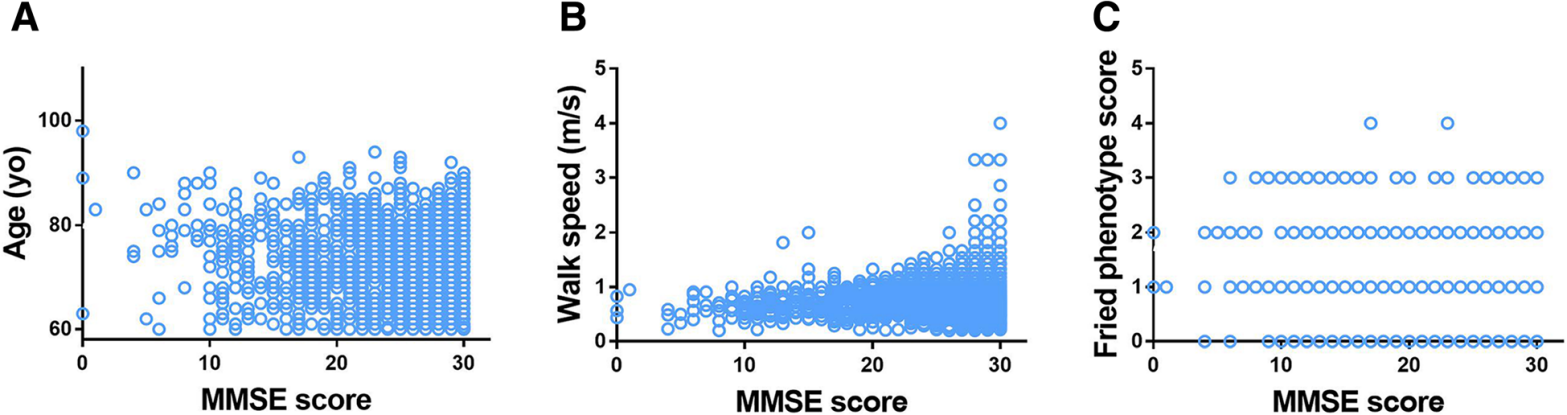
Correlation between MMSE total score with age and frailty. Spearman’s rank correlation test was used to determine the relationships between MMSE total score with age (**a**), walking speed (**b**), and Fried phenotype score (**c**) among adults aged 60 years and older in China by CCGAS, 2011–2012. The total sample population in the analysis was 3202

Table 2 shows the effect of frailty on cognition. Frail participants performed worse on global cognition and all the ten domains than both robust and prefrail participants. Prefrail residents scored statistically less in the areas of global cognition, reading, drawing, writing, repetition, orientation to time, orientation to place, recall, and attention than robust older adults. We further conducted correlation between the MMSE score and many variables; the MMSE score was positively correlated with walking speed (*r* = 0.244, *p* < 0.001) and negatively correlated with age (*r* = − 0.2426, *p* < 0.001), and frailty phenotype score (*r* = − 0.2835, *p* < 0.001) (Fig. 1).

**Table 2:** The effect of frailty on cognition

	Robust (*n* = 1798)	Prefrail (*n* = 1087)	Frail (*n* = 317)
MMSE total score	27.54 ± 3.72	26.46 ± 4.86*	22.79 ± 6.59*Δ
Comprehension	2.92 ± 0.32	2.87 ± .045	2.70 ± 0.67*Δ
Reading	0.96 ± 0.19	0.94 ± 0.23*	0.84 ± 0.37*Δ
Naming	1.99 ± 0.13	1.99 ± 0.14	1.95 ± 0.30*Δ
Drawing	0.74 ± 0.44	0.69 ± 0.46*	0.48 ± 0.50*Δ
Writing	0.97 ± 0.20	0.94 ± 0.24*	0.84 ± 0.37*Δ
Repetition/registration	2.90 ± 0.40	2.80 ± 0.51*	2.61 ± 0.79*Δ
Orientation to time	4.66 ± 0.89	4.46 ± 1.15*	3.66 ± 1.67*Δ
Orientation to place	4.80 ± 0.66	4.69 ± 0.85*	4.20.291 ± 1.35*Δ
Recall	2.52 ± 0.79	2.39 ± 0.89*	1.93 ± 1.10*Δ
Attention	5.09 ± 1.59	4.69 ± 1.85*	3.58 ± 2.19*Δ

^*^P < 0.05, compared to robust group; ^Δ^*P* < 0.05, compared to prefrail group

A multivariate logistic regression revealed that after adjusting for age, gender, education level, living area, and chronic diseases, frailty [hazard ratio (HR): 2.571(1.789–3.695), *p* < 0.001], exhaustion [HR: 2.099(1.389–3.172), *p* < 0.001], slowness [HR: 1.859(1.327–2.606), *p* < 0.001], and inactivity [HR: 1.709(1.250–2.335), *p* = 0.001] were significantly associated with global cognition. Weight loss was not independently associated with cognition (Table 3).

**Table 3:** Multivariate logistic regression of frailty components on global cognition

Frailty component	Model 1		Model 2		Model 3	
	HR (95% CI)	P	HR (95% CI)	P	HR (95% CI)	P
Frailty	5.076(3.750–6.871)	< 0.001	4.270(3.117–5.851)	< 0.001	2.571(1.789–3.695)	< 0.001
Weight loss	1.942(1.176–3.206)	0.010	1.695(1.016–2.827)	0.043	–	0.103
Exhaustion	3.346(2.392–4.680)	< 0.001	2.759(1.953–3.899)	< 0.001	2.099(1.389–3.172)	< 0.001
Slowness	2.406(1.821–3.179)	< 0.001	2.081(1.562–2.771)	< 0.001	1.859(1.327–2.606)	< 0.001
Inactivity	2.762(2.105–3.623)	< 0.001	2.654(2.013–3.499)	< 0.001	1.709(1.250–2.335)	0.001

Model 1: not adjusted

Model 2: adjusted for age and gender

Model 3: model 2 adjusted for education, living area and chronic diseases

## Discussion

In the nationwide survey, we found the standard overall prevalence of physical frailty, prefrailty, CI, and CF in Chinese older adults to be 8.8, 33.8, 6.5, and 2.0%, respectively, which was in accordance with previous studies [18, 21]**.** Recent studies have shown the prevalence of physical frailty, CI, and CF to be 5.1, 5.5, and 1.1%, respectively, in Japanese older adults [22], and the prevalence of CF was 4.4% in Italian older adults [23]. A recent review estimated the prevalence of CF to be as low as 1.0–1.8% in community settings [24], where it is associated with a high risk of disability, dementia, poor quality of life, and death [22–24]. The aforementioned studies suggest that CF might be a useful concept for clinicians and a potentially preventive or therapeutic target for dementia and disability in later life.

We found that frail participants had worse global cognition in all the domains of the MMSE than both robust and prefrail participants, and prefrail residents scored statistically lower in the areas of global cognition, reading, drawing, writing, repetition, orientation to time, orientation to place, recall, and attention than robust older adults. Other studies also found worse cognitive functioning in frail patients, controlling for age and gender [23]; furthermore, robust but cognitively impaired participants are more likely to be prefrail/frail than normal cognitive participants [25], which indicates that CI might take part in the pathogenic mechanisms of frailty. Frailty was also associated with metacognitive executive dysfunction [26]. There is a cumulative but no interactive effect of frailty and CI in the prediction for mortality [27].

We further found that the MMSE total score was positively correlated with walking speed and negatively correlated with age and frailty total score. A multivariate logistic regression revealed that after adjusting for age, gender, education level, living area, and chronic diseases, global cognition was significantly lower in participants with frailty, exhaustion, slowness, and inactivity. A previous study had found that an increase in age is accompanied by a cognitive decline with decreased grip strength, slower walking speed, and more severe depression [28]. A recent review revealed that physical activity was associated with changes in executive function and word recall and global cognitive function was associated with grip strength, walking speed, and exhaustion [29]. Physical frailty is a stronger indicator of everyday and global cognition than age [30]; both baseline status and within-person changes in frailty were predictive of cognitive trajectories [10]. The information process speed and decline rate over time may have an important role in the onset of frailty [31]. Slow walking speed was associated with CI [32]. After controlling for other variables, each 0.10 increase in baseline frailty was associated with a 0.01 increase in CI at follow-up, while each 0.1 increase in baseline CI was associated with a 0.003 increase in frailty at follow-up [33]. In another study, although frailty was associated with poor baseline cognitive performance, there were no effects on slopes of cognition, suggesting that frailty was not associated with cognitive decline, which might be explained by the fact that frailty-related cognitive deficits may exist independent of mechanisms underpinning neurodegenerative disorders [34], providing further evidence of the importance of the notion of CF.

Given that early-stage intervention may be more effective in older adults and that the frailty continuum is reversible [35], prefrailty, an intermediate and preclinical state, should be included in the concept of CF. We found that the prevalence of the combination of prefrail/frail and CI was 5.1% (weighted 4.5%). Another study found that prefrailty combined with lower cognition scores at baseline was associated with higher risks of poor quality of life, incident physical limitation, increased cumulative hospital stay, and mortality [25]. Prefrailty was associated with worse memory and processing speed [36]. Exercises may delay or reverse cognitive decline, indicating that early detection of CF has public health implications [37].

To date, there is no consensus on the definition of CF or on how CI and physical frailty should be measured. Numerous frailty tools make an operational definition of CF and the development of preventive intervention strategies more difficult. For example, a study found that the prevalence of frailty was 7.5% using the Edmonton Frail Scale but 30% using CHS criteria indicating that the identification of frailty in CI is partly dependent on assessment methods [38]. A systematic review identified seven methods of cognitive assessment in frailty operationalization such as dementia as comorbidity and objective cognitive screening instruments [39]. Delayed recall, language, and praxis used as criteria for CI in combination of prefrailty can predict adverse outcomes [25]. Very recently, Yu et al. found that the use of a single cognitive domain may be effective in characterizing CI groups, and that the use of prefrailty also identifies a subset of individuals at risk of progressing to frailty [25]. In this study, we chose the Fried phenotype for frailty assessment and the MMSE for cognition measurement, which were easy to use in clinical work and for large populations. We further included the prefrailty in the construct of CF, which provided the evidence that prefrailty in the CF in Chinese older adults.

The present study has several limitations. First, the cross-sectional design made it difficult to interpret the cause-effect relationship of the association between physical frailty and CI. Second, we used a four-item version of the Fried criteria, as weakness (measured by grip strength) was not considered in the survey and weight loss was defined as low BMI, rather than as a quantified change in weight over time. Third, cognition was assessed by the MMSE instead of a battery of neuropsychological measurements. Brain imaging data were lacking in the current study. However, our study also has several strengths. The CCGAS, a nationwide survey in China, is based on well-established cluster, stratified, and random selection statistical sampling techniques, and the seven cities were representative of the six main regions in China. Furthermore, we provided the first empirical epidemiological evidence of the prevalence of CF based on the Fried phenotype in mainland China.

## Conclusion

Our study provides epidemiological evidence of the prevalence of physical frailty, prefrailty, CI, and CF in Chinese older adults as well as the association between physical frailty and CI. Frailty and its components except weight loss were independently associated with CI. Combined with the growing epidemiological evidence from other countries, physical frailty is linked to CI and the concept of CF provides a new approach to prevent later-life function decline and dementia. However, the mechanisms underlying the relationship between physical frailty and CI, as well as CF should be further studied.

## Abbreviations

ADL: Activities of daily living
BMI: Body mass index
CCGAS: China Comprehensive Geriatric Assessment Study
CF: Cognitive frailty
HR: Hazard ratio
IADL: Instrumental activities of daily living
MCI: Mild cognitive impairment
MMSE: Mini-Mental State Examination
WHR: Waist-to-hip ration
